# Interrelationship among Obstructive Sleep Apnea, Renal Function and Survival: A Cohort Study

**DOI:** 10.3390/ijerph17144922

**Published:** 2020-07-08

**Authors:** Patrizia Pochetti, Danila Azzolina, Beatrice Ragnoli, Paolo Amedeo Tillio, Vincenzo Cantaluppi, Mario Malerba

**Affiliations:** 1 Respiratory Unit, Sant’ Andrea Hospital, 13100 Vercelli, Italy; patrizia.pochetti@tin.it (P.P.); beatrice.ragnoli@hotmail.it (B.R.); paoloamedeo.tillio@uniupo.it (P.A.T.); 2 Department of Traslational Medicine, University of Eastern Piedmont, 28100 Novara, Italy; danila.azzolina@uniupo.it; 3 Department of Traslational Medicine, Nephrology and Kidney Transplant Unit, University of Eastern Piedmont, 28100 Novara, Italy; vincenzo.cantaluppi@uniupo.it

**Keywords:** obstructive sleep apnea, C-PAP, creatinine, eGFR

## Abstract

Previous studies showed a bidirectional relationship between renal function decline and obstructive sleep apnea (OSA) syndrome. Continuous Positive Airway Pressure (C-PAP) treatment was shown to preserve the kidney function in OSA patients. This study aims to investigate the progression of long-term renal function in OSA patients treated with different PAP strategies (patients were divided into two groups, fixed C-PAP or other PAP—automatic and bilevel pressure). Comorbidities and 10-years survival were also evaluated. We performed a retrospective, observational, single-center, cohort study, including the first 40 consecutive patients enrolled from 2009 in the Respiratory disease Unit at the Vercelli University Hospital database. The patient inclusion criteria were: age ≥ 18 years with OSA syndrome according to AASM (American Academy of Sleep Medicine) guidelines. Creatinine serum levels (mg/dL) and the estimated Glomerular Filtration Rate (eGFR, mL/min calculated by CKD-EPI (Chronic Kidney Disease Epidemiology Collaboration equation)) were measured at 3 different time points: at baseline, 3 years and 8 years after PAP treatment. The Kaplan–Meier survival curves stratified according to PAP treatment and compliance have been reported together with log-rank test estimation. In our study, we found a significant creatinine serum level reduction after 3 years of fixed C-PAP treatment (*p* value = 0.006) when compared to baseline values. However, we observed that the long-term C-PAP benefit was not significant (*p* value = 0.060). Our data confirmed the progressive renal function decline in OSA patients, especially in those using other-PAP treatments; nevertheless, OSA treatment with a fixed C-PAP device has shown, in the short term, a significant improvement in renal function. By contrast, in our study, long-term benefits after 8 years are not been demonstrated probably because of the lack of compliance of the patients and the aging effect.

## 1. Introduction

Renal function decline is a massive economic problem [1]. Chronic Kidney Disease (CKD) prevalence has been continuously increasing in the last years [1], and it has been associated with an enhanced risk of cardiovascular morbidity and death [2]. Diabetes, hypertension, and obesity are the most common causes of CKD [3]. However, these chronic comorbidities do not completely explain the increase in CKD prevalence [4].

Previous studies showed a bidirectional relationship between renal function decline and obstructive sleep apnea (OSA) syndrome. OSA is the most common kind of sleep apnea characterized by upper airway collapse during sleep with repeated episodes of apneas and hypopneas leading to oxygen desaturations [5,6,7,8,9,10,11]. OSA is related to congestive heart failure, hypertension, stroke, arrhythmias, and worsens the prognosis of coronary artery disease [12,13,14,15,16,17]. The organ damage is related to intermittent hypoxia, inducing oxidative stress, inflammatory state, hemodynamic instability, and increased sympathetic activity; all of these factors contribute to tissue injury that can be mainly ascribed to endothelial dysfunction [18].

It was estimated that more than 17% of the general population suffers from OSA [12,13,14,15,16,17,18,19], but the prevalence of OSA in CKD patients has been reported to be even greater than 40% [5].

Kidneys are sensitive to hypoxia and they suffer from intermittent desaturation related to sleep apnea [20]. Other authors described a significant association between OSA related hypoxemia and a faster decline of kidney function: intermittent hypoxia can damage the kidney through oxidative stress disorder [9,21]. Some studies showed a correlation between nocturnal hypoxemia in OSA patients and a worsening of kidney function mediated by reactive oxygen species, the direct activation of the nervous system, the activation of the renin-angiotensin system [5,22,23,24,25,26,27,28,29,30]. These dangerous agents may induce glomerular hyperfiltration, resulting in proteinuria and a consequent decline in glomerular filtration [31,32].

The beneficial effects of Continuous Positive Airway Pressure (C-PAP) therapy on cardiovascular diseases are well known [33]. C-PAP treatment according to some authors appeared to also protect OSA patients’ kidney function: retrospective, observational cohort studies reported that OSA treatment with C-PAP improved the short-term kidney function [8,22], showing, in particular, a significant association between the presence of OSA and glomerular filtration rate in C-PAP treatment [22]. More than an improvement of glomerular hyperfiltration reducing nocturnal hypoxia in sleep apnea patients with C-PAP [31], it was demonstrated that C-PAP therapy in OSA subjects was associated with improved renal hemodynamics and the down-regulation of renal renin-angiotensin activity, suggesting a potential therapeutic benefit on kidney function [5].

The aim of this study was to investigate the progression of long-term renal function data in adult OSA patients enrolled in Vercelli Register according to polysomnographic data, different PAP strategy treatment (patients were divided into two groups, fixed C-PAP or other PAP (Positive Airway Pressure)—automatic and bilevel pressure), comorbidities and 10-years survival focusing on patient characteristics. The treatment effects have been evaluated considering as primary outcome variables serum creatinine and estimated Glomerular Filtration Rate (eGFR) values after PAP intervention and after 8 years of follow-up.

## 2. Methods and Patients

### 2.1. Study Design and Setting

This study was conducted in agreement with the STROBE statement for observational studies [34]. We performed a retrospective, observational, single-center, cohort study, including the first 40 consecutive patients enrolled from 2009 in the Vercelli Register in Respiratory Unit at the Vercelli University Hospital (Department of Translational Medicine, University of Eastern Piedmont) with a diagnosis of OSA confirmed by polysomnographic study according to American Academy Sleep Medicine (AASM) diagnostic criteria [35]. All study participants voluntarily signed an informed consent form. The study was conducted in accordance with the Declaration of Helsinki, and the protocol was approved by the local ethic committee (2018-2497SMP).

### 2.2. Patients and Samples

Inclusion criteria were: age ≥ 18 years with OSA syndrome according to AASM guidelines [35] included from 2009 in Vercelli Register (40 consecutive patients).

We performed polysomnography (Embletta TM; Vitalnight TM, Natus Medical Incorporated, Pleasanton, CA, USA) for the OSA diagnosis. The apneas were defined as more than a 90% reduction in the amplitude of nasal flow for at least 10 s, and hypopneas as more than a 30% reduction in the amplitude of nasal flow for at least 10 s associated with at least a 4% reduction in oxyhemoglobin saturation. C-PAP machines were able to reach pressure from 4 to 20 cmH2O; all PAP machines had an internal memory card for the data storage.

For each patient, we recorded anthropometric data: age, sex, BMI, smoking habit (current or former), cardiovascular, respiratory and metabolic comorbidities, polysomnographic data at baseline and during PAP treatment (Apnea-Hypopnea Index, AHI, Oxygen Desaturation Index (ODI), Nocturnal Median Oxygen Saturation, time under 90% of Oxygen Saturation – t90), PAP treatment mode and pressure levels (fixed C-PAP, auto C-PAP or Bilevel), renal function (serum creatinine levels and eGFR with CKD-EPI equation) at 3 different points (baseline, 3-years after and 8-years after), PAP treatment adherence data (obtained by medical reports) and 10-years survival. The patient compliance with PAP treatment (fixed C-PAP and other-PAP devices) was evaluated extracting medical reports obtained at the same time as renal function assessment and recorded in our hospital database.

A patient was defined as compliant to the therapy if a PAP use of more than 4 h and 5/7 nights use was demonstrated [35].

Medical reports recorded, for compliant patients, a PAP treatment adherence duration of more than 4 h in the night for at least 5 nights in the week. The therapeutic outcomes on OSA parameters using PAP were: AHI < 5 and ODI < 5 with a therapeutic pressure well-tolerated by patients. C-PAP therapy was started in all OSA patients, but some of those did not tolerate the adequate therapeutic pressure able to reach the therapeutic outcomes on OSA parameters, for this reason, according to good medical practice, if C-PAP (first-choice-therapy) was not tolerated, we started a different PAP therapy according to the patients referred problems, using automatic PAP or Bilevel to reach normalizations of AHI and ODI goals. According to this method, the C-PAP sample was larger than the other-PAP group, but the two groups were similar in terms of age, sex, and renal function at baseline.

Renal function measurements were retrospectively investigated from the Vercelli Hospital Database (Galileo TM, Hitech, La Muela, Spain). We collected serum creatinine levels (mg/dL) and an estimated Glomerular Filtration Rate (eGFR mL/min): eGFR was calculated with the CKD-EPI equation [36]. Creatinine and eGFR were measured at 3 different time points after starting PAP treatment, and we calculated the delta values during treatment compared to baseline.

### 2.3. Sample Size

A sample size of 40 subjects is needed to ensure a power of 0.8 to detect a Cohen standardized delta effect size [37] of 0.8 (with a significance level of 0.05) in the creatinine delta between C-PAP and other interventions. The creatinine variation in the C-PAP group is assumed to be equal to 0.05 (SD = 0.029) [21]. A two-sided T-test for the difference in means has been considered for the computation.

### 2.4. Statistical Analysis

Descriptive statistics of data in the two PAP groups have been performed. The data were reported as median (I and III quartiles) for continuous variables, and percentages (absolute numbers), for qualitative variables. The Wilcoxon–Kruskal–Wallis test was performed for continuous variables and the Pearson chi-square test was performed for categorical ones.

Descriptive statistics for the distribution of serum creatinine and eGFR endpoints were reported. *p*-values for Wilcoxon paired samples have been adjusted by the multiplicity of testing across time point comparisons using the Benjamini and Hochberg method [38].

According to potential creatinine and eGFR correlation, a simultaneous outcome assessment has been performed via the multiple marginal GEE (Generalized Estimating Equation) model [39,40]. The multiple marginal models have been estimated considering both creatinine and eGFR scales as simultaneous outcomes; a 95% confidence interval has been computed on estimated effects.

The Kaplan–Meier survival curves stratified according to PAP treatment and compliance have been represented together with log-rank test estimation.

Statistical analysis has been performed using the R System version 3.6.2 [41] and mmmgee [42], gee [43], multcomp [44], survival [45], and rms [46] packages (R Systems, El Dorado, CA, USA).

## 3. Results

Patients’ baseline characteristics are shown in Table 1. All patients underwent PAP treatment. Thirty-one subjects used C-PAP devices, the remaining tested other PAP modalities (automatic C-PAP or Bilevel, for C-PAP treatment failure or intolerance). The 80% of our cohort was composed of males with a mean age of 59 years. The patients were predominantly obese (BMI > 30) with moderate-to-severe OSA disease severity accordingly to AASM criteria [35] (Table 1).

We did not identify baseline significant differences between the two groups (Table 1). Moreover, we did not observe any significant difference in renal function outcome according to serum creatinine and eGFR delta values in the two PAP groups before starting PAP therapy (Table 2).

In our sample, a significant reduction in serum creatinine level after 3 years of C-PAP therapy was observed. Conversely, after 8 years of follow-up, the protective effect of continuous PAP treatment on renal function is still present, but not significant (Table 3). Concerning the eGFR outcome, we observed a significant deterioration after 8 years (Table 3). No significant variations in the creatinine serum level and eGFR are evidenced in the other-PAP group.

A significant increase in serum creatinine and the consequent reduction of eGFR levels were observed in elderly patients according to the decline in renal function for aging.

The long-term follow-up benefit of treatment on renal function was not confirmed by the multivariable model, which showed a significant increase of serum creatinine levels in male patients; moreover, eGFR worsened in association with the increase in age and smoking habits (Table 4).

### 3.1. Polysomnographic Data

C-PAP therapy was started in all OSA patients, but some of those did not tolerate the adequate pressure to reach the therapeutic outcomes on OSA parameters, so, according to good medical practice, if C-PAP (first-choice-therapy) was not tolerated, we started a different PAP therapy according to the patients referred problems, using automatic PAP or Bilevel to obtain normalizations of AHI (Apnea-Hypopnea Index) and ODI. All treatments were assessed with Polysomnography (PSG), and the results are shown in Table 5. All treatments were successful at PSG after PAP titration, but this result had to be maintained with adequate compliance, as evaluated in the text. The most important reason for C-PAP intolerance was the impossibility to maintain for at least 4 h at night the C-PAP therapy set at the correct level of therapeutic pressure—continuous pressure may be discomfortable for some patients, in this case providing a different PAP modality we could avoid to discontinue PAP treatment. 

In the C-PAP treatment group, the median ODI before treatment (Table 3) was 24 (48, 16); the median value of the time under 90% of Oxygen Saturation (t90) was 12 (38, 4).

We considered polysomnographic data among patients (*n* = 12; 30% of the whole sample) characterized by the worsening of serum creatinine levels after an 8-year of follow-up. One patient (8.3%) had AHI < 30 at baseline, the others suffered from severe OSA.

### 3.2. Compliance to Positive Air Pressure Treatment

Among patients with the worsening of serum creatinine after 8 years, we observed a 50% lack of adherence to the PAP treatment. By contrast, in the group of patients with stable renal function (showing a reduction of serum creatinine levels after 8 years), 82.2% was compliant to the PAP device.

### 3.3. 10 Years Survival

The overall mortality rate after ten years of follow up was 25% (80% males, 90% smokers, 30% Chronic Obstructive Pulmonary Disease (COPD), 60% diabetics, 80% hypertensive patients). Of note, 50% of deaths occurred in other-PAP treatment (Auto C-PAP and Bilevel) group; after 10 years, 62.5% of OSA patients receiving a therapy different from fixed C-PAP were dead.

**Figure 1:**
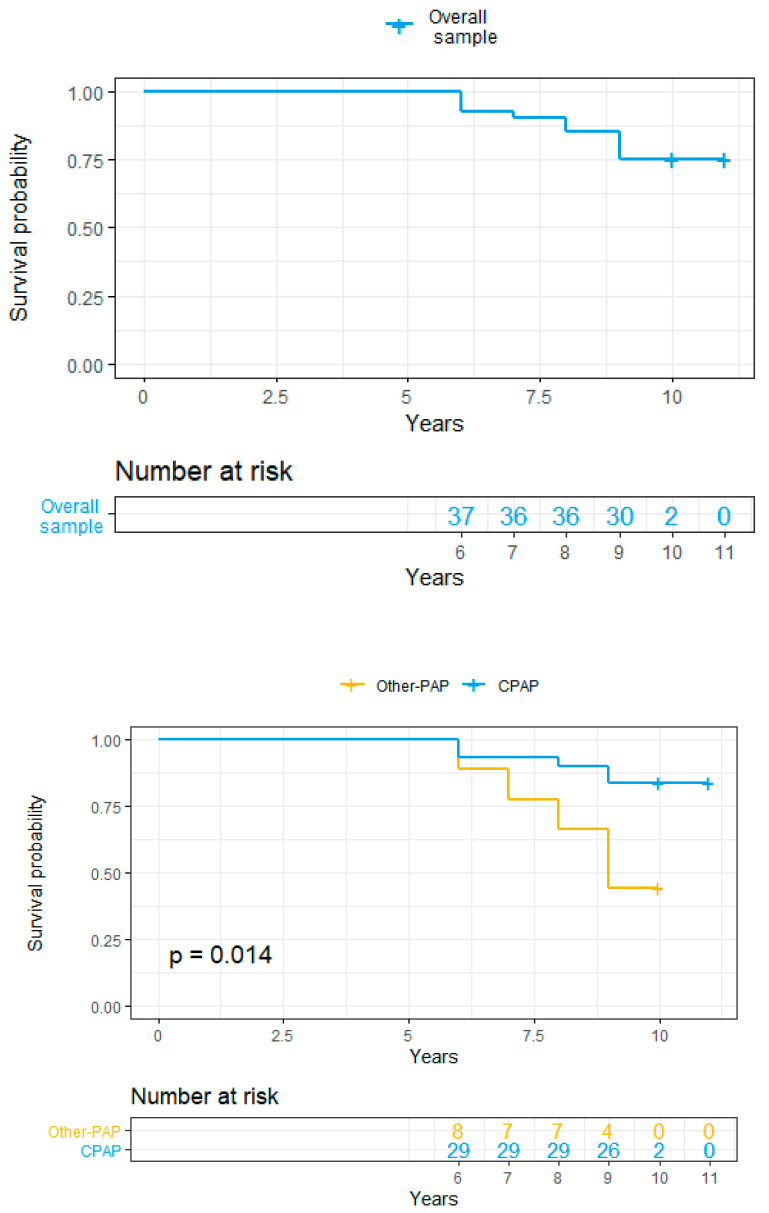

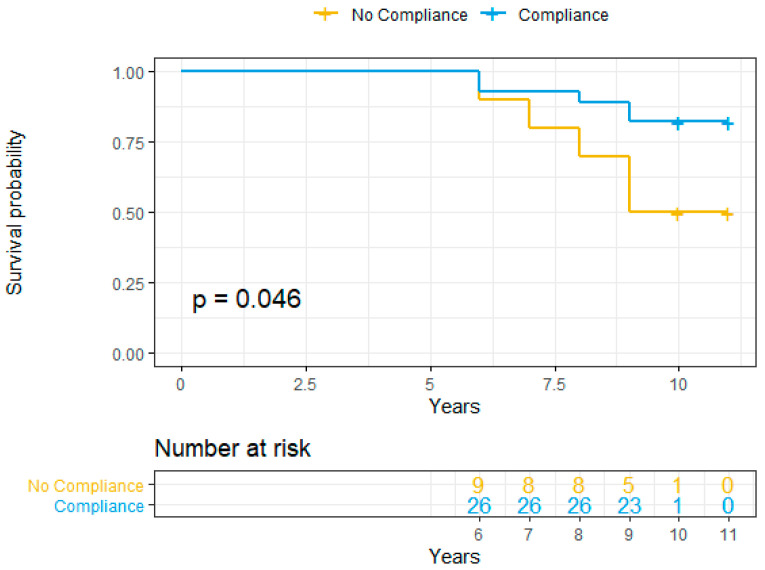
Kaplan–Meier Survival Curves overall and accordingly to PAP therapy and compliance. C-PAP = Continuous Positive Airway Pressure; PAP = Positive Airway Pressure.

The 10 years survival rate was significantly higher in patients with a better compliance (Figure 1).

## 4. Discussion

The results of the present study cohort have shown that short-term fixed C-PAP treatment improved renal function in OSA patients. The short-term worsening of renal function was prevented in the C-PAP group. Fixed C-PAP treatment, according to other findings, is probably superior to other-PAP devices to obtain the improvement of renal function [8,22,25], even if a limited sample size was available for other-PAP therapy.

The short-term positive effects of C-PAP on renal function may be ascribed to a limitation of ROS (Reactive Oxygen Species) induced tissue damage and particularly to the deactivation of the renin–angiotensin–aldosterone system (RAAS) and of the autonomous nervous system that modulate glomerular filtration. However, consistently with the literature [16,22,47], we found a general deterioration of renal function in the long-term follow-up (8 years), especially in the patients having a limited compliance with the device. After 8 years of follow-up, serum creatinine levels were higher in severe patient accordingly to baseline AHI; among patients with a deterioration of serum creatinine levels after 8 years a high number of nocturnal desaturations (baseline ODI) was also observed.

Our results suggest that the long-term renal function decline may occur in severe OSA patients despite PAP therapy. This aspect, in our cohort, may be related to the lack of compliance, the severity of disease at diagnosis accomplished with a long-term exposure to the toxic effect of apnoea and desaturation finally leading to organ damage. Moreover, the disease severity at diagnosis, male sex, smoking habits, obesity, diabetes, hypertension, COPD, were factors characterizing an increased mortality after 10 years (25% of the whole sample).

Our observations are consistent with the literature: OSA is a major risk for cardiovascular and metabolic diseases [12,13,14,15,16,18] and mortality [48]. Decreased renal function was associated with both obstructive and central sleep apnea; there is a 20–40% increased risk of OSA in patients with CKD [8,22]. OSA is also likely to contribute to developing CKD or to enhance its progression toward end stages with the need of dialysis once it is established [22]. Factors that may contribute to the OSA-associated deterioration of renal function include: intermittent hypoxia and kidney tubular damage and endothelial dysfunction, association with diabetes and hypertension; a worsening in the renal function might also contribute to worsening OSA, increasing upper airway edema; metabolic acidosis may lead to a subsequent ineffective respiratory compensation that globally promotes a pH reduction [23]. OSA in CKD patients may go underdiagnosed, because patients overlapping for these two conditions are less likely to be overweight or snore, and their fatigue may be attributed to uremia [8].

The present study also provides evidence that several confounding factors may affect CKD progression; for example, a worsening of serum creatinine and eGFR levels was demonstrated in elderly patients, because ageing is related to structural and functional changes of the kidney, even in the absence of other comorbidities [49,50]. For example, a reduction in kidney cortical volume may be shown by an increase in the amount and size of simple renal cysts. Moreover, all the microstructural and histologic nephrosclerosis signals increase in elderly patients. Another aging-related disease, such as hypertension or arterial stiffness, is related to a deterioration of renal function [51].

The present study also shows that kidney health status is a sex-related factor, since an increase in serum creatinine was observed more in males in comparison to females [52,53].

Furthermore, lifestyle factors may be associated with kidney health status in OSA patients [27]. For example, smokers and OSA patients had a more evident increase of eGFR in comparison to nonsmokers. Another study evidence was that smoking habit was harmful to the kidneys, leading to an increased risk to develop proteinuria [8]. Several causes may be responsible for proteiniuria in this cohort of patients, including endothelial dysfunction with a consequent alteration of glomerular permeselectivity. Moreover, experimental and clinical studies clearly demonstrated that one of the main determinant of CKD progression is the loss of tubular epithelial cell function with consequent atrophy and interstitial fibrosis. We could speculate that hypoxia associated with a poor compliance for C-PAP may contribute to CKD progression and to an increased incidence of low molecular weight proteinuria [8].

Taken together, the results of the present study suggest that, in OSA patients, an inadequate C-PAP compliance associated with different comorbidities and aging lead to a long-term decline in kidney function that is probably due to the decrease of Renal Functional Reserve (RFR): RFR is defined as the capacity of the kidney to increase GFR in the presence of different stimuli. The progressive loss of RFR predisposes to Acute Kidney Injury (AKI) episodes and to a faster GFR decline; of note, in aging OSA patients with chronic comorbidities, RFR can be reduced in the presence of normal GFR values [54].

The present study has some limitations. For example, serum creatinine and eGFR have intrinsic variability that may prevent the possibility to detect a variation between the C-PAP and other-PAP.

Analysis of this data will provide a measurement in each patient of the variability in these indices of kidney function over several months before enrolment. In addition, we have several measurements of eGFR and ACR after enrolment, which will reduce the chance of a single sporadic change confounding our analysis.

This is a pilot study to obtain preliminary data useful to determine the feasibility of a larger study, and further researches are needed to investigate long-term renal function and survival of OSA patients.

GFR measurement by creatinine clearance evaluation might improve the analysis of renal function in comparison to GFR estimation by the CKD-EPI (Chronic Kidney Disease Epidemiology Collaboration equation formula). Unfortunately, in this retrospective analysis we did not have samples of 24-hr urine collection. Moreover, we have to consider that creatinine clearance reflects only glomerular filtration, whereas a part of OSA-associated renal dysfunction may be related to hypoxic tubular injury. In this setting, a new generation of urine biomarkers able to detect tubular damage may improve the diagnosis of epithelial-to-mesenchymal transition, leading to fibrosis and CKD progression in association with GFR and RFR data [55]. The novelty of this study consists of the possibility to evaluate a long-term effect of different PAP treatment on renal function, highlighting the patient characteristics that may lead to renal function decline and long-term survival. These results are useful to give preliminary indications for the physicians to tailor the OSA therapy on the patient giving more attention to the elderly subject and their comorbidities.

Moreover, educational sessions and repeated follow-up visits may be also considered in combination with C-PAP therapy as well as patient behaviors (smoking, obesity, compliance to C-PAP therapy) are relevant aspects affecting the renal function decline and survival.

## 5. Conclusions

Our data suggest a progressive renal function decline in OSA patients in the long-term follow-up (8 years) especially in patients with reduced compliance to the PAP device or a treatment different from fixed C-PAP.

OSA treatment with a fixed C-PAP device has shown a significant improvement in renal function in the short term, indicating a protective role of C-PAP therapy on renal function decline in OSA patients.

Long-term benefits after 8 years are not consistent with our results according to the lack of compliance and the aging effect. In the group of patients with a decline in renal function after 8 years, we observed a worse polysomnographic outcome at diagnosis. In our data, a 25% 10-year mortality rate has been assessed, especially among males, smokers, and patients affected by obesity, diabetes, hypertension, and COPD.

We underline the importance of phenotyping OSA patients according to their specific characteristics (age, compliance, behavioral aspect) and comorbidities, factors likely contributing to renal function decline and long-term survival.

## Figures and Tables

**Table 1:** Descriptive table on the baseline characteristics accordingly to the intervention group.

Variable	Other-PAP	C-PAP	Combined	*p*-value
	(*n* = 9)	(*n* = 31)	(*n* = 40)	
Sex: Female	22% (2)	19% (6)	20% (8)	0.800
Male	78% (7)	81% (25)	80% (32)	
Age at Diagnosis	63 (58, 70)	60 (50, 67)	60 (54, 68)	0.300
BMI	33 (31–35)	33 (30, 36)	33 (30, 35)	0.700
Smoker: No	22% (2)	29% (9)	28% (11)	0.700
yes	78% (7)	71% (22)	72% (29)	
AHI at diagnosis	36 (28, 36)	28 (16, 32)	30 (19, 35)	0.500
Diabetes: No	44% (4)	68% (21)	62% (25)	0.200
Yes	56% (5)	32% (10)	38% (15)	
CHD: No	89% (8)	90% (28)	90% (36)	0.900
Yes	11% (1)	10% (3)	10% (4)	
CVD: No	89% (8)	100% (31)	98% (39)	0.060
Yes	11% (1)	0% (0)	2% (1)	
COPD No	78% (7)	84% (26)	82% (33)	0.700
Yes	22% (2)	16% (5)	18% (7)	
Asthma: No	100% (9)	77% (24)	82% (33)	0.100
Yes	0% (0)	23% (7)	18% (7)	

Continuous data are reported as median (I, III quartiles), categorical data are reported as absolute numbers (percentage). BMI = Body Mass Index; CHD = Coronary Heart Disease; CVD = Cardio-Vascular Disease; COPD = Chronic Obstructive Pulmonary Disease; C-PAP = Continuous Positive Airway Pressure; PAP = Positive Airway Pressure; *n* = Number.

**Table 2:** Descriptive table of outcome variations accordingly to the intervention group.

Variable	Other-PAP	C-PAP	Combined	*p*-Value
	(*n* = 9)	(*n* = 31)	(*n* = 40)	
Creatinine delta (after−3 years)	0.03 (−0.12, 0.13)	0.08 (0, 0.16)	0.08 (0, 0.16)	0.300
Creatinine delta (after−8 Years)	0.005 (−0.207. 0.225)	0.015 (−0.108. 0.135)	0.015 (−0.118, 0.157)	0.900
eGFR delta (after−3 years)	22 (22, 22)	−0.5 (−6, 5.8)	2 (−5, 9)	0.100
eGFR delta (after−8 Years)	10 (4, 15)	4 (−4, 8)	4 (−4, 8)	0.500

Continuous data are reported as median (I, III quartiles), categorical data are reported as absolute numbers (percentage). C-PAP = Continuous Positive Airway Pressure; PAP = Positive Airway Pressure; *n* = Number.

**Table 3:** Descriptive table of outcome in C-PAP (Panel 1) and other-PAP data (Panel 2).

Panel 1 C-PAP Treatment			
**Variable**	**Baseline**	**3 Years**	***p*** **-Value**
	(*n* = 31)	(*n* = 31)	
Creatinine	1 (0.9, 1.1)	0.9 (0.8, 1)	0.006
eGFR	88 (73, 97)	93 (79, 101)	0.920
**Variable**	**Baseline**	**8 Year**	***p*** **-Value**
	(*n* = 31)	(*n* = 31)	
Creatinine	1 (0.9, 1.1)	0.9 (0.8, 1.1)	0.620
eGFR	88 (73, 97)	79 (64, 94)	0.880
**Variable**	**3 years**	**8 Year**	***p*** **-Value**
	(*n* = 31)	(*n* = 31)	
Creatinine	0.9 (0.8, 1)	0.9 (0.8, 1.1)	0.060
eGFR	93 (79, 101)	79 (64, 94)	0.049
**Panel 2 Other-PAP Treatment**			
**Variable**	**Baseline**	**3 Years**	***p*** **-Value**
	(*n* = 31)	(*n* = 31)	
Creatinine	0.8 (0.8, 0.9)	0.7 (0.6, 1)	0.900
eGFR	76 (74, 77)	101 (74, 115)	0.730
**Variable**	**Baseline**	**8 Year**	***p*** **-Value**
	(*n* = 31)	(*n* = 31)	
Creatinine	0.8 (0.8, 0.9)	0.8 (0.5, 2.0)	0.910
eGFR	76 (74, 77)	100 (78, 108)	0.700
**Variable**	**3 years**	**8 Year**	***p*** **-Value**
	(*n* = 31)	(*n* = 31)	
Creatinine	0.7 (0.6, 1.0)	0.8 (0.5, 2.0)	0.920
eGFR	101(74, 115)	100 (78, 108)	0.850

Data are reported as median (I-III quartiles). *p*-values for Wilcoxon paired samples have been adjusted by the multiplicity of testing using the Benjamini and Hochberg method [33]. eGFR (Glomerular Filtration Rate); C-PAP = Continuous Positive Airway Pressure; PAP = Positive Airway Pressure; *n* = Number.

**Table 4:** Estimates, 95% confidence interval, and a *p*-value for the multiple GEE model.

Outcome		*β*-Effect	95% CI	*p*-Value
Creatinine	Age	0.011	[−0.001; 0.02]	0.082
	BMI	−0.006	[−0.028; 0.02]	0.572
	Follow up time – 3 years vs. baseline	−0.044	[−0.136; 0.05]	0.349
	8 year vs. baseline	0.117	[−0.102; 0.34]	0.295
	treatment - other-PAP vs. C-PAP	−0.002	[−0.576; 0.57]	0.994
	Sex – Male	0.257	[0.034; 0.48]	0.024
	Active Smoker –Yes	0.133	[−0.059; 0.32]	0.174
eGFR	Age	−1.155	[−1.922; −0.39]	0.003
	BMI	0.141	[−1.175; 1.46]	0.834
	Follow up time –3 years vs. baseline	0.18	[−8.819; 9.18]	0.969
	8 year vs. baseline	−10.61	[−19.924; −1.29]	0.026
	treatment - other-PAP vs. C-PAP	8.972	[−15.899; 33.84]	0.480
	Sex –M	4.002	[−9.783; 17.79]	0.569
	Smoke –Yes	−13.34	[−26.68; 0]	0.050

Multiple GEE (Generalized Estimating Equation) models creatinine and eGFR (Glomerular Filtration Rate) simultaneously, thus accounting for mutual correlation among them. C-PAP = Continuous Positive Airway Pressure; PAP = Positive Airway Pressure; *n* = Number; BMI = Body Mass Index.

**Table 5:** Polysomnographic data.

Variable	Other-PAP	C-PAP	Combined	*p*-Value
	(*n* = 9)	(*n* = 31)	(*n* = 40)	
AHI (Basal)	36 (28, 36)	28 (16, 32)	30 (19, 35)	0.500
AHI (3-years)	0 (0, 0)	1 (0, 2)	1 (0, 2)	0.300
AHI (8-years)	15 (15, 15)	2 (2, 3)	3 (2, 9)	0.300
ODI (Basal)	30 (30, 30)	24 (16, 48)	26 (16, 44)	0.600
ODI (3-Years)	7 (5,8)	2 (1, 3)	2 (1, 4)	0.050
ODI (8-Years)	19 (19, 19)	10 (9, 10)	10 (10, 14)	0.300
T90 (Basal)	7 (7, 7)	12 (4, 38)	11 (5, 38)	0.800
T90 (3-Years)	1.2 (1.1, 1.3)	1 (0.3, 2)	1 (0.6, 1.7)	0.700
T90 (8-Years)	3 (3, 3)	4 (3, 6)	3 (2, 6)	0.999
Mean Nocturnal Oxygen Saturation (%) (Basal)	94 (94, 94)	92 (91, 92)	92 (92, 92)	0.100
Mean Nocturnal Oxygen Saturation (%) (3-Years)	95 (95, 96)	95 (94, 95)	95 (94, 95)	0.300
Mean Nocturnal Oxygen Saturation (%) (8-Years)	94 (94, 94)	94 (93, 94)	94 (94, 94)	0.700

C-PAP = Continuous Positive Airway Pressure; PAP = Positive Airway Pressure; *n* = Number; AHI (Apnea-Hypopnea Index); ODI = Oxygen Desaturation Index.
